# A Correlation Driven Approach with Edge Services for Predictive Industrial Maintenance

**DOI:** 10.3390/s18061844

**Published:** 2018-06-05

**Authors:** Meiling Zhu, Chen Liu

**Affiliations:** 1 School of Computer Science and Technology, Tianjin University, Tianjin 300350, China; 2 Beijing Key Laboratory on Integration and Analysis of Large-Scale Stream Data, North China University of Technology, Beijing 100144, China; liuchen@ncut.edu.cn; 3 Institute of Data Engineering, North China University of Technology, Beijing 100144, China

**Keywords:** sensor data, event correlations, proactive data service, service hyperlink, edge computing

## Abstract

Predictive industrial maintenance promotes proactive scheduling of maintenance to minimize unexpected device anomalies/faults. Almost all current predictive industrial maintenance techniques construct a model based on prior knowledge or data at build-time. However, anomalies/faults will propagate among sensors and devices along correlations hidden among sensors. These correlations can facilitate maintenance. This paper makes an attempt on predicting the anomaly/fault propagation to perform predictive industrial maintenance by considering the correlations among faults. The main challenge is that an anomaly/fault may propagate in multiple ways owing to various correlations. This is called as the uncertainty of anomaly/fault propagation. This present paper proposes a correlation-based event routing approach for predictive industrial maintenance by improving our previous works. Our previous works mapped physical sensors into a soft-ware-defined abstraction, called proactive data service. In the service model, anomalies/faults are encapsulated into events. We also proposed a service hyperlink model to encapsulate the correlations among anomalies/faults. This paper maps the anomalies/faults propagation into event routing and proposes a heuristic algorithm based on service hyperlinks to route events among services. The experiment results show that, our approach can reach 100% precision and 88.89% recall at most.

## 1. Introduction

Predictive industrial maintenance aims at enabling proactive scheduling of maintenance, and thus minimizing unexpected device faults. Nowadays, predictive industrial maintenance is well studied when data deviate from normal behavior within individual sensors in recent years [1]. However, a fault is not always isolated. Due to the obscure physical interactions, trivial anomalies will propagate among different sensors and devices, and gradually deteriorate into a severe fault [2]. Mining such propagation paths is an effective method for predictive industrial maintenance.

We will examine a typical scenario below. In a coal power plant, there are hundreds of devices running continuously and thousands of sensors have been deployed. From individual sensors, anomalies, i.e., values deviating from normal behaviors, can be regarded as events [3]. Such events correlate with each other in multiple ways across sensors and devices. These correlations uncover possible propagation paths of anomalies among different devices. They are very helpful in explaining the root cause of an observable anomaly/fault and perform predictive industrial maintenance proactively.

**Figure 1:**
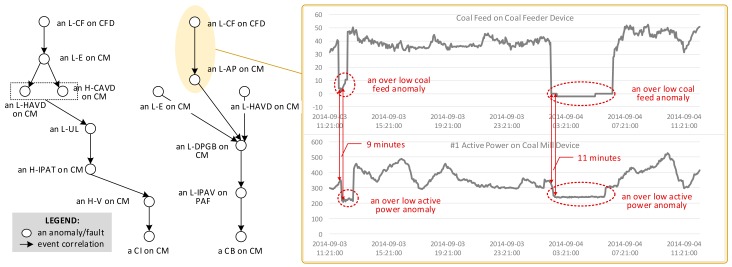
Partial anomaly propagation under correlations among sensors and devices in a coal power plant.

For clarity, we list all abbreviations used in this scenario in Table 1. Figure 1 illustrates partial propagation paths starting from the L-CF. We find three observations from this scenario.

The first observation is that anomalies/faults are correlated with each other and evolve into a severer one along these correlations. Taking the case on the right-hand side of Figure 1 as an example, an L-CF is usually followed by an L-AP. In other words, an L-CF and an L-AP always occur together in order within [9min, 11min]. We regard the L-CF as the cause of the occurrence of L-AP. Along the causal relationship, an L-CF propagates into an L-AP from the CF sensor to the AP sensor.

Secondly, an anomaly/fault can be affected by others in two ways. Sometimes, an anomaly/fault can be caused by the co-occurrence of several anomalies/faults. As shown in the left case of Figure 1, an L-HAVD and an H-CAVD leads to an L-UL. The absence of any of them will not cause an L-UL. Besides, an anomaly/fault can be caused separately by several anomalies/faults. For example, the occurrence of any anomaly/fault in L-E, an L-AP, and an L-HVAD may cause the L-DPGB. The two ways of causing an anomaly/fault should be considered during anomaly/fault propagation.

The third observation from the scenario is that an anomaly/fault may propagate in multiple ways owing to different correlations. Due to space limitation, Figure 1 only shows the partial propagation paths starting from the L-CF. An L-CF can propagate into different faults, such as a CI fault and a CB fault, which are both a severe fault on a coal mill device. This is called as the uncertainty of anomaly/fault propagation.

The observations show that causal relationships among anomalies/faults provide us lots of clues for depicting propagation paths. Thus, the first issue is to mine these correlations for laying foundation for depicting the propagation paths. The uncertainty of anomaly/fault propagation is another problem we have to deal with in making predictive industrial maintenance.

Based on these observations, this paper tries to perform industrial maintenance proactively by predicting the anomalies/faults propagation by using the correlations. Our previous works laid foundation of this attempt.

In our previous works, we proposed a service-based framework to dynamically correlating the sensor data and generating higher-level events between sensors and applications [4,5,6]. We mapped physical sensors into a software-defined abstraction deployed in the cloud, called proactive data service. A proactive data service takes data from sensors and events from other services as inputs and transforms them into new events based on user-defined operations. Also, we proposed a new abstraction, called service hyperlink, to encapsulate relationships between the services. With service hyperlinks, a service can dynamically route an event to the other services at runtime. Our previous works enables us to depict the anomaly/fault propagation as the event routing among services.

However, our previous works cannot be easily put in to practice. Firstly, public cloud infrastructure is not suitable for a power plant since its data can reveal many significant factors such as national economic situation and energy consumption. On the other hand, a power plant is deployed with more than ten thousand sensors. A private cloud has a limitation of scale and scalability to handle such amount of data. Edge computing paradigm provides a good opportunity to handle the problem.

Secondly, our previous works paid more attention on measuring the strength of positive relationship between two sensors from a statistical perspective. However, the above observations show that the casual relationships among anomalies/faults can help a lot to propagate anomalies and faults, which is that an anomaly/fault always happens following some other ones while the corresponding sensors may be not correlated at all.

The present paper tries to propose a heuristic approach to route events among services via service hyperlinks by improving our previous works. It uses the event routing path to predict the anomalies/faults propagation among sensors and perform predictive industrial maintenance. To reach this goal, its main contributions include: (1) It tries to refine our proactive data service model to fit edge computing paradigm. In this way, our approach can be applied in information-sensitive industrial enterprises; (2) It focus on a new kind of causal relationship among anomalies and faults and transform the correlation mining problem into a time-constrained frequent co-occurrence pattern mining problem. We propose an effective algorithm to mine the correlations and encapsulate them into service hyperlinks; (3) This paper develops a heuristic event routing approach to handle the uncertainty issue. (4) We try validate the completeness of our approach by model checking techniques, i.e., whether any anomaly/fault propagation path which exists in an industrial system can be captured by the graph. Besides, we do rich experiments to verify our approach based on a real dataset and several synthetic datasets in a coal power plant.

## 2. Related Works

### 2.1. Predictive Industrial Maintenance

Predictive industrial maintenance is a hot topic in mechanical engineering. Professor Legutko, S. and his team considered that predictive maintenance provided new opportunities to reduce the operational cost on equipment replacements and increase enterprises’ economy [7,8,9]. Recent predictive maintenance techniques can be classified into three categories, model-based approaches, data-driven approaches, and hybrid approaches [10,11,12,13,14,15,16,17,18,19].

Model-based approaches usually build models on training datasets based on prior knowledge and verify the model on testing datasets. Vianna, W.O.L. et.al. proposed a method to identify degradation and their future estimates and then integrate their results into a maintenance planning optimization algorithm for aeronautical redundant systems based on extended Kalman filter [10]. Jung, D. et al. propose a new analytical framework and a new data analytic engine supporting Remaining Usefulness Lifetime (RUL) estimation. Their analysis algorithm exploited the new feature generation scheme to build reliable models over meaningful feature domain. It can be used to identify different equipment classes with completely different ageing model [11]. Simões, A. et al. describes two algorithms that can help to increase the quality of assessment of engine states and the efficiency of maintenance planning based on a hidden Markov model [12]. Wang, J. et al. presented a general classification-based failure prediction method. Their parameterized model systematically defined four categories of features to cover all possibly useful features, and then used feature selection to identify the most important features for model construction [13].

On the other hand, data-driven approaches take no account of prior knowledge and build models based on data completely. Patil, R.B. et al. presented an overall methodology of predicting failure of these devices by using data-driven approach based on machine learning, for reducing machine downtime, improving customer satisfaction and cost savings for original equipment manufacturers [14]. Sipos, R. et al. presented a data-driven approach based on multiple-instance learning for predicting equipment failures. The approach mined equipment event logs usually not designed for predicting failures but contain rich operational information [15]. Susto, G.A. et al. presented an adaptive flexible maintenance scheduling decision support system. The proposed system employed Machine Learning and regularized regression methods to refine remaining useful life estimates [16]. Some researches focused on detect anomalies from individual sensors by data-driven techniques to address predictive industrial maintenance. Sammouri, W. et al. proposed a methodology for the prediction of infrequent target events in temporal sequences. They transformed data sequences into a data matrix and decreased the number of attributes by removing less significant and contributive attribute. Then, they applied pattern recognition classifiers to predict the occurrence of infrequent target events [17]. Bezerra, C.G. et al. proposed a comparative analysis of three recently introduced outlier detection methods for fault detection applications [18].

Some researches combine the data-driven techniques with model-based approaches. They used these techniques to learn parameters of models and update models with the movement of time. Baptista, M. et al. proposed a framework that can predict when a component/system will be at risk of failure in the future, and therefore, advise when maintenance actions should be taken. Authors employed an auto-regressive moving average (ARMA) model along with data-driven techniques to facilitate the prediction [19]. Liao, W. et al. developed a hybrid machine prognostics approach to predict machine’s health condition and describe machine degradation. Based on machine’s prognostics information, a predictive maintenance model was well constructed to decide machine’s optimal maintenance threshold and maintenance cycles [20].

All these predictive industrial maintenance approaches lay foundation of our work. However, almost all these techniques construct a model based on prior knowledge or data in the build-time. Neither of them considers the changing of the internal relationships among anomalies/faults. This paper makes an attempt on predictive industrial maintenance from the novel perspective.

### 2.2. Edge Computing

Edge computing aims at bringing back partial computation load from the cloud to the edge devices. Edge can be regarded as any computing resources along the path between data sources and data center. Smart phones can be the edges between body sensors and cloud. Researchers propose a system which is named MAUI [21]. The system decides at runtime which methods should be remotely executed in a smart phone to achieve the best energy savings while minimizing the changes required to applications. Smart gateway is another choice as an edge between home sensors and cloud in a smart home scenario. In literature [22], authors propose a system, which abstracts connected entities as services and allows applications to orchestrate these services with end-to-end QoS requirements. They illustrate the system by a health smart home use-case. In the case, a gateway is an edge node and maintains services. A laptop can also be an edge node sometimes. Researchers present Vigil, a real-time distributed wireless surveillance system supporting real-time tracking and surveillance in enterprise campuses, retail stores, and across smart cities. Vigil utilizes laptops as edge computing nodes between cameras and cloud to save wireless capacity [23]. Besides, some people place edge comping nodes on the cloud. In literature [24], authors virtualize a physical sensor as a virtual sensor on the cloud and automatically provisioning the virtual sensors on demand. In our research, we borrow ideas from Vigil [23] and utilize laptops as edge computing nodes. Each computing node can maintain multiple proactive data services and transmit generated event streams to the data center.

### 2.3. Service Relationship

Service relationship has attracted much attention in the field of service computing. Dong et al. tried to capture the temporal dependencies based on the amounts of calls to different services [25]. Hashmi et al. proposed a framework for web service negotiation management based on dependency modeling for different QoS parameters among multiple services [26]. Wang et al. considered that a dependency is a relation between services wherein a change to one of the services implies a potential change to the others [27]. They utilized a service dependency matrix to solve the service replacement problem.

However, most of the existing work only considers input/output dependency, pre/post condition dependency, correlations among services and so on. Neither of them takes the dependency of the involved data/events.

### 2.4. Event Relationship

#### 2.4.1. Event Correlation

Existing studies of event correlation are the foundation of our work. Event correlation discovery is a hot topic [28,29,30,31,32,33,34,35]. It can be used in various areas like process discovery [28,29,30,31], anomaly detection [32,33], healthcare monitoring [34,35] and so on. In the field of business process discovery, event correlation challenge is well known as the difficulty to relate events that belong to the same case. Pourmirza et al. proposed a technique called correlation miner, to facilitate discovery of business process models when events are not associated with a case identifier [28,29]. Cheng et al. proposed a new algorithm called RF-GraP, which provides a more efficient way to discover correlation over distributed systems [30]. Reguieg et al. regarded event correlation as correlation condition, which is a predicate over the attributes of events that can verify which sets of events belong to the same instance of a process [31]. Some studies used event correlation to detect anomalies. Friedberg et al. proposed a novel anomaly detection approach. It keeps track of system events, their dependencies and occurrences, and thus learns the normal system behavior over time and reports all actions that differ from the created system model [32]. Fu et al. focused on temporal correlation and spatial correlation among failure events. They developed a model to quantify the temporal correlation and characterize spatial correlation. Failure events are clustered by correlations to predict their future occurrences [33]. Other works applied event correlation in healthcare monitoring. Forkan et al. concentrated on vital signs, which are used to monitor a patient’s physio-logical functions of health. The authors proposed a probabilistic model to make predictions of future clinical events of an unknown patient in real-time using the learned temporal correlations of multiple vital signs from many similar patients [34,35].

#### 2.4.2. Event Dependency

Recently, some researchers focus on event dependencies. Song et al. mined activity dependencies (i.e., control dependency and data dependency) to discover process instances when event logs cannot meet the completeness criteria [36]. In that paper, the control dependency indicates the execution order and the data dependency indicates the input/output dependency in service dependency. A dependency graph is utilized to mine process instances. However, the authors do not consider the dependency among events. Plantevit et al. presented a new approach to mine temporal dependencies between streams of interval-based events [37]. Two events have a temporal dependency if the intervals of one are repeatedly followed by the appearance of the intervals of the other, in a certain time delay.

## 3. Proactive Data Service Model

### 3.1. Preliminaries

This paper aims at proposing a correlation-based event routing approach to predict anomalies/faults propagation among sensors at the software layer. However, most of current physical sensors produce sensor data. They do not afford configurability and programmability so that cannot generate and route events. Therefore, we need an abstraction of these sensors at the software layer. Our previous works [4,5,6] proposed a proactive data service model, which is the abstraction and lays foundation for this paper.

Although lots of studies have focused on how to encapsulate sensor data into services, the service models intrinsically follow the “request-and-response” manner [38,39,40,41,42,43,44]. We previously proposed a novel type of service abstraction, called proactive data service, with which we hope to find a more automatic and quick way for handling sensor data and events from other services while maintaining the common data service capabilities. A proactive data service can autonomously respond to all events it receives. Relationships among services are encapsulated into service hyperlinks to facilitate the event routing among services: When an event generated from a service, it will be routed into other services via hyperlinks. The services receiving the event will be stimulated to respond to it in the same manner.

In our previous works, when building a service, a user customizes its functionality by customizing the input sensor data as well as operations. Event handler invokes operations for different inputs. Event definition is responsible for defining output event type and format. In this way, each service processes its inputs and generates high-level events. A created service can be encapsulated into a Restful-like API so that other services or applications can use it conveniently. As mentioned above, service hyperlink is an important component for routing generated events.

### 3.2. Proactive Data Service Model Refinement

Our proactive data service model was proposed to encapsulate sensor data generated in industrial environments. The formal definition of sensor data is presented below.

**Definition** **1.** *(Sensor Data): A piece of sensor data is a 3-tuple: d = (timestamp, sensorid, value), in which timestamp is the generation time of d; sensorid represents the sensor generates d; value is the value of d.*

Example (Sensor Data): A piece of sensor data *d* = (2014-09-03 11:21:00, A6, 31.356) represents that the CF sensor (id: A6) generates a value of 31.356 at 2014-09-03 11:21:00.

A time-ordered list of sensor data generated from a same sensor forms a sensor data sequence. Figure 1 shows two examples of sensor data sequence, including a CF sensor sequence, and an AP sensor sequence.

We next discuss how to refine the service model. Placing all proactive services in the cloud to serve real industrial applications in information-sensitive enterprises is impractical. A public cloud is not suitable for information-sensitive enterprises because of data privacy. On the other hand, a privacy cloud cannot afford complete functionalities due to its limited resources and capacities. Edge computing paradigm provides a good opportunity to handle the issue. To fit edge computing paradigm, we try to place our proactive data service in edge nodes to bring back partial computation load from the cloud. Such service is called as an edge proactive data service or an edge service for short. The limitation of edge nodes limits the functionality of an edge service. Therefore, each edge service is refined to encapsulate sensor data from one sensor. It is responsible for detecting sensor data deviating from most one by user-defined operations, i.e., anomalies, and encapsulating them as events. These events are more valuable than sensor data but much fewer than sensor data. To avoid complex computation, an edge service receives no event from other services, and thus its event handler is not in use. Other components, including event definition and service hyperlink, retain in edge services. Besides, the edge service can also be encapsulated into a Restful-like API.

Our approach is proposed to depict anomalies/faults propagation. Thus, edge services detect anomalies and send them to the cloud is reasonable. This can also avoid sending redundant sensor data so that can relieve the transmission load of the network and the computation pressure in the cloud.

There are many traditional techniques and algorithms can be borrowed to detect anomalies, such as range-based approaches, outlier detection approaches and discord discovery approaches. A range-based algorithm customizes value bounders for individual sensors based on inspectors’ experiences, sensor/device instructions and so on. Outliers are widely known as the values which sufficiently deviate from the most ones. A discord is the subsequence which are most dissimilar with others in a sequence. There are many excellent works with open source code on these topics (An open source of an outlier detection software: https://elki-project.github.io/; an open source of a discord discovering technique: http://www.cs.ucr.edu/~eamonn/MatrixProfile.html.). With these techniques, an edge service can detect anomalies from individual sensors. These anomalies can be regarded as events by many studies [3]. This paper follows them and considers them as service events.

**Definition** **2.** *(Service Event): A service event, which also refers to a service event instance or an instance, is a 4-tuple: e = (timestamp, eventid, serviceid, type), in which timestamp is the generation time of e; eventid is the unique identifier of e; serviceid is the unique identifier of the service generating e; and type is the type of e.*

Example (Service Event): The L-CF in Figure 1 can be expressed as a service event *e* = (2014-09-04 02:24:00, 11686, S6, L-CF). It represents an over low coal feed service event (id: 11686) occurring at 2014-09-04 02:24:00, which is generated by the CF service (id: S6).

A service event sequence is a time-ordered list of service events generated by a same service. Here is an example of a service event sequence in Figure 1: *E* = 〈(2014-09-03 12:00:00, 11685, S6, L-CF), (2014-09-04 02:24:00, 11686, S6, L-CF)〉.

To depict anomaly/fault propagation, we also need the details of each fault in an industrial system. These can be extracted from maintenance records.

**Definition** **3.** *(Maintenance Record): A maintenance record is a 4-tuple r = (rid, start_time, end_time, fault_desc), where rid is the record id, start_time and end_time is the starting time and ending time of this fault, and fault_desc is the text for fault description.*

Example (Maintenance Record): For example, there is a maintenance record *r* = (116,928, coal blockage, 2014-09-04 04:21:00, over low inlet primary air volume in #2 coal mill: coal blockage in #2 coal mill). The over low inlet primary air volume is the sign of coal blockage fault.

This paper tries to use event routing among services to depict anomalies/faults propagation. The cloud needs to undertake this task based on our previous works. Therefore, we also preserve some proactive data services in the cloud, which is called as cloud proactive data service or cloud service for short. Different from edge services, each cloud service encapsulates a fault in an industrial system. In this way these cloud services can provide an opportunity to depict the anomalies/faults propagation.

A fault can be identified by a non-empty set of anomalies. Existing techniques such as association rules can help to do this [45]. These anomalies are generated from edge services. Consequently, a service should take the anomalies, i.e., service events from the corresponding edge services as its inputs for identifying the fault this cloud service encapsulates. It also receives service events from other cloud services for routing events. The event handler is indispensable for our cloud services. Other components are the same with edge services. Based on the above analysis, we give the formal definition of the refined proactive data service.

**Definition** **4.** *(Proactive Data Service): A proactive data service is defined as an 8-tuple: s_i_ = (uri, APIs, input_channels, event_handler, operations, event_definition, output_channel, service_hyperlinks). uri is the unique identifier; APIs is a set of RESTful-like APIs; input_channels represents a set of channels receiving different kinds of inputs; event_handler invokes different operations for different input service events; operations is a set of operations used for processing the inputs; event_definition is responsible for defining out-put service event type and format; output_channel represents the channel for outputting service events generated by operations; service_hyperlinks is essentially a routing table, which can point out the target services of each output service event. Proactive data service can be categorized into two types:*

- *edge service: the service model for encapsulating sensor data from one sensor, where event_handler = ϕ, and input_channels is used for receiving sensor data.*
- *cloud service: the service model for encapsulating a fault, where input_channels s used for receiving service events.*

Our refined service model decouples the sensor data from analysis. The edge services encapsulate sensor data and can customize valuable service events for tasks in the cloud. This simple functionality in edge nodes is for relieving the load of the network and cloud. In predictive industrial maintenance, the two kinds of services provide a richer layered view of the anomaly/fault propagation. The cloud services show the faults interactions macroscopically. The edge services present the root causes of a fault. A user can switch between the two layers conveniently.

## 4. Service Hyperlink Model

Based on the scenario at the beginning, the causal relationships among anomalies/faults play an important role in anomalies/faults propagation. As a result, our service hyperlink is an abstraction of causal relationships among service events. It reflects the causes of each service event. The causes and this event forms a pattern. Formally, let E = {*E*_1_, …, *E_k_*} be *k* service event sequences, E^t^ be a set of service event types in *E*_1_, …, *E_k_*, and there exists e*_i_*^t^ ∈ E^t^, E_-_^t^ = E^t^−{e*_i_*^t^}, 〈E_-_^t^, {e*_i_*^t^}〉 becomes a time-constrained frequent co-occurrence pattern, short for TFCP, if the following conditions are satisfied: (1) instances of E^t^ occur together *f*(〈E_-_^t^,{ e*_i_*^t^}〉) times, *f*(〈E_-_^t^,{e*_i_*^t^}〉)≥δ_co_, where δ_co_ is a times threshold; (2) instance of e*_i_*^t^ has the largest timestamp in each occurrence of Et. In a TFCP 〈E_-_^t^, {e*_i_*^t^}〉, E_-_^t^ is called as antecedent, and e*_i_*^t^ is called as consequent. Figure 1 implies an example of a TFCP: 〈{L-CF}, {L-AP}〉.

Obviously, there is a causal relationship between the E_-_^t^ and e*_i_*^t^ in a TFCP. Many studies measure the relationship by the conditional probability *p*(e*_i_*^t^|E_-_^t^) = *f*(〈E_-_^t^,{e*_i_*^t^}〉)/*f*(E_-_^t^), where *f*(〈E_-_^t^,{e*_i_*^t^}〉) and *f*(E_-_^t^) are the occurrence times of 〈E_-_^t^,{e*_i_*^t^}〉 and E_-_^t^ respectively [46].

Besides, anomaly/fault propagation always occurs within a time interval. As shown in Figure 1, an L-CF anomaly propagates into an L-AP anomaly in 9 to 11 min. The time interval is also an important information to depict the propagation and should be considered in the definition of service event correlation. According to the service event correlation, we can refine service hyperlink.

**Definition** **5.** *(Service Event Correlation): Let E_-_^t^ be a set of service event types and e_i_^t^ be another type, there is a service event correlation between E_-_^t^ and e_i_^t^ if and only if 〈E_-_^t^,{e_i_^t^}〉 is a TFCP. The service event correlation is denoted as γ(E_-_^t^, e_i_^t^) = (E_-_^t^, e_i_^t^, T_int_, p), where E_-_^t^ is the causes, e_i_^t^ is the effect, T_int_ = [t_min_, t_max_] is the propagation time interval, and p is the conditional probability.*

Example (Service Event Correlation): Figure 1 shows a service event correlation ({L-CF}, {L-AP}, [9min, 11min], 1.0). It means that an L-CF anomaly is followed by an L-AP anomaly in 9 to 11 min with 100% chance.

**Definition** **6.** *(Service Hyperlink): Let γ(E_-_^t^, e_i_^t^) be a service event correlation, where E_-_^t^ are contained by a set S of proactive data services, e_i_^t^ is contained by a service s_i_. Given a probability threshold δ_p_, if p ≥ δ_p_, there is a service hyperlink L(S, s_i_) = (S, s_i_, γ(E_-_^t^, e_i_^t^), δ_p_), where S is a set of source services, s_i_ is the target service.*

Service hyperlinks encapsulate valuable service event correlations, i.e., the ones with high enough probability.

## 5. Service Hyperlink Generation

### 5.1. Problem Analysis

This section discusses how to generate service hyperlinks. Service events are generated and sorted by time into service event sequences. From these sequences, we mine service event correlations and encapsulate them into service hyperlinks.

Based on definition 5, the service event correlation *γ*(E_-_^t^, e*_i_*^t^) is measured by a conditional probability. To calculate the probability, we have to count the occurrence times *f*(〈E_-_^t^,{e*_i_*^t^}〉) of 〈E_-_^t^,{e*_i_*^t^}〉 in an event sequence set. Besides, the co-occurrence time interval of 〈E_-_^t^,{e_i_^t^}〉 needs to be recorded as propagation time interval of the service hyperlink. Thus, the task of mining service event correlations is easily transformed into mining TFCPs with recording co-occurrence time interval.

The challenge of TFCP mining is that a TFCP 〈E_-_^t^,{e*_i_*^t^}〉 consists of two event type groups, where intra-group’s instances (instances of E_-_^t^) are unordered and inter-group’s instances are time-ordered (instances of E_-_^t^ occur earlier than instance of e*_i_*^t^). Traditional frequent co-occurrence pattern mining algorithms cannot directly handle the challenge. They only focused on the occurrence frequency of a group of unordered objects [47,48]. But they still give an inspiration to us for developing an effective algorithm to mine TFCPs.

### 5.2. Service Event Correlation Generating

#### 5.2.1. Frequent Co-Occurrence Pattern Mining

This section reminds the concept of traditional frequent co-occurrence pattern and the techniques of mining such patterns.

We list some related concepts. A group of objects *O* = {*o*_1_, *o*_2_, …, *o_k_*} from a sequence *E_i_* is a co-occurrence pattern, if max{*T*(*O*)}-min{*T*(*O*)}, where *T*(*O*) = {*t_o_*_1_, *t_o_*_2_, …, *t_ok_*}, *t_oj_* is the occurrence time of *o_j_* (*j* = 1, 2, …, *k*) in *E_i_*, and ∆*t* is a predefined time threshold. The co-occurrence pattern *O* becomes a frequent co-occurrence pattern, if it occurs in no less than *δ* sequences.

Researchers tried to generate all co-occurrence patterns and count them to discover frequent ones [47,48].

#### 5.2.2. TFCP Mining

Our task is to mine all TFCPs whose occurrence times are no less than *δ_co_*. All TFCPs can be grouped by its consequent, i.e., *R* = ⋃*R*(e^t^), where *R* is the complete set of TFCPs, *R*(e^t^) = {〈E_-_^t^,{e*_i_*^t^}〉| e*_i_*^t^ = e^t^∧〈E_-_^t^,{e*_i_*^t^}〉 is a TFCP}. Each group can be mined separately in the service event sequence set E = {*E*_1_, *E*_2_, …, *E_k_*}. Such divide and conquer strategy has been widely used in frequent pattern mining problem [49].

Firstly, we generate potential consequents by computing the occurrence times of each event type in a service event sequence set E = {*E*_1_, …, *E_k_*} by *f*(e^t^) = ∑*_i_n_i_*, where *n_i_* is the occurrence times of e^t^ in sequence *E_i_*. Service event types whose occurrence times are no less than *δ_co_* are selected as potential consequents, which is denoted as *C_cq_*.

For each service event type e^t^ in *C_cq_*, we generate the corresponding TFCP set *R*(e^t^) separately. Every type e_j_^t^ (e*_j_*^t^ ≠ e^t^) in *C_cq_* will be selected to generate a potential TFCP 〈{e*_j_*^t^},{e^t^}〉. Then we test whether 〈{e*_j_*^t^},{e^t^}〉 is a TFCP with consequent e^t^ by judging whether *f*(〈{e*_j_*^t^},{e^t^}〉) ≥ *δ_co_*. During this process, we record the co-occurrence time interval. After generating a valid TFCP, we extend it by adding a third type e*_k_*^t^ ∈ *C_cq_* (e*_k_*^t^ ≠ e*_j_*^t^, e*_k_*^t^ ≠ e^t^) into the antecedent. We test whether 〈{e*_j_*^t^, e*_k_*^t^},{e^t^}〉 is a TFCP with consequent e^t^ in the same manner. The extension is repeated until there is no new valid TFCP. There is a skill during the extensions to avoid generating repeated TFCPs, i.e., all types are added in lexicographical order. It indicates that we only add a larger service event type to a validated TFCP. Thus, we can easily mine the service event correlations in generated TFCPs.

#### 5.2.3. Service Hyperlink Generating

A service event correlation will be encapsulated into a service hyperlink if *p* ≥ *δ_p_*, where *δ_p_* is a probability threshold. Details of service event correlation encapsulation can be found in our previous works [5,6].

## 6. Our Predictive Industrial Maintenance Approach

### 6.1. The Framework of Our Approach

**Figure 2:**
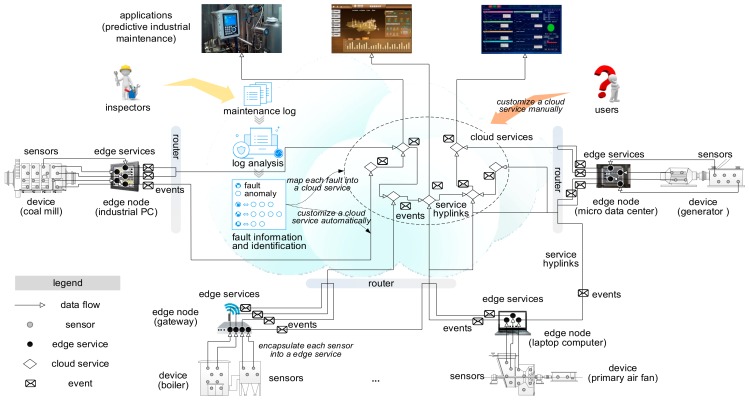
The framework of our approach.

Based on the refined proactive data service and service hyperlink, we propose a service hyperlink-based approach to route service events among services for predictive industrial maintenance. Figure 2 presents the framework. Our approach plugs the gap between sensors and applications. Edge services at the edge side abstract sensors at the software layer. Edge services can provide a unified interface for heterogeneous sensor data and make sensor data accessible easily. They enhance the flexibility and reusability of sensor data sources. Besides, an edge service is preset with anomaly detection operations to make simple analysis on input sensor data. It encapsulates detected anomalies into service events and send them into the cloud instead of sensor data. In the cloud, our event routing approach facilitate service events route among cloud services and applications. Once a service event is generated, our approach will compute the most probable destination it may reach and the most probable path to the destination. The service event will be routed in this way. And if the probability exceeds a threshold, the service will also send this event to applications for planning predictive maintenance.

### 6.2. Proactive Data Service Graph Generating

**Figure 3:**
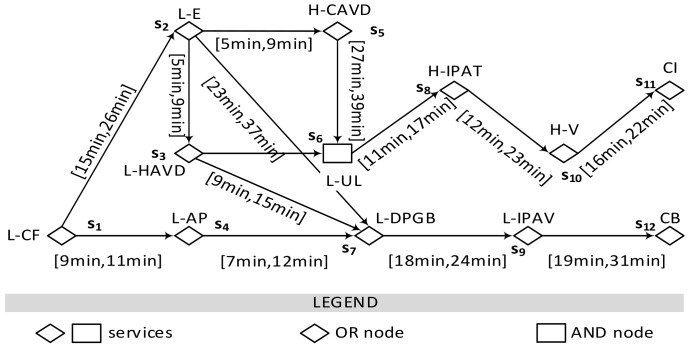
An example of Proactive Data Service Graph (PDSG).

To route service events among services, we first need to generate a graph formed by proactive data services and service hyperlinks. This graph is proposed as a way to describe the anomalies, faults, and their consequences over time. It is a directed graph model where its node represents an edge/cloud service, which generates service events. A node corresponding to a cloud service is categorized into two types to reflect the two ways of causing a fault mentioned in our scenario. An edge is the service hyperlink encapsulating causal relationships between service events. Each edge is labeled with propagation time interval. The graph is formally defined as follows. Figure 3 illustrates an example of a Proactive Data Service Graph (PDSG) following Figure 1.

**Definition** **7.** *(Proactive Data Service Graph, PDSG): A PDSG is a directed graph G = 〈V, E〉, where:*

- *V = A∪F, F is the complete set of edge proactive data services, and F is the complete set of cloud proactive data services. Each node v ∈ F should be AND type or OR type. AND type implies that the fault represented by v occurs if all anomalies and faults pointing to v occur; OR type implies that the fault represented by v occurs if any anomaly or fault pointing to v occurs.*
- *E ⊆ V × F is a non-empty edge set. Each edge e ∈ E is labelled with a propagation time interval T_int_.*

Number of generated service hyperlinks can help to determine the AND/OR type of a node. We denote *SHL*(*α*) = {*L*(S,*s_i_*)|e*_i_*^t^ = *α*} be all service hyperlinks whose effect event is *α*. Thus, these service hyperlinks have same target service, which is written *s_α_*. If |*SHL*(*α*)| > 1, the cloud service *s_α_* is defined as an OR node. Otherwise, *s_α_* is defined as an AND node.

### 6.3. Event Routing on Proactive Data Service Graph

In a PDSG, services are connected with each other intricately. The destination of a service event is uncertain during its routing. This paper uses a heuristic approach to handle the problem.

Firstly, as the destination of a service event is uncertain, we try to find all its possible destinations and compute the most probable one it may reach in the future as its destination. In a PDSG, a service *s_i_* can reach a service *s_j_*, i.e., *s_j_* is reachable from *s_i_*, if there exists a sequence of adjacent services (i.e., a path) which starts from *s_i_* and ends with *s_j_*. Based on the definition of PDSG, all services reachable from a given service *s_i_* are cloud services. Therefore, when a service *s_i_* generates a service event, all reachable services may be the destinations of this service event. However, the number of these destinations may be too large. For example, as Figure 3 shows, an L-CF service event from *s*_1_ may be routed to each rest service on the graph. Finding the routing path to all potential destinations are too expensive. Furthermore, it may cause repeat maintenance plans. For instance, an L-IPAV and a CB fault are generated by service *s*_9_ and *s*_12_ on one path. If people predict that a CB fault is going to happen, they will certainly realize that an L-IPAV will happen before the CB fault. But if the L-IPAV is stopped, the CB will not happen. Therefore, there is no need to plan the maintenance twice for the two faults respectively. Consequently, a candidate destination set is needed to be selected from all potential destinations. Herein, a candidate destination set can be regarded as these reachable services which are not on the path to other reachable services. The formal definition is shown below.

**Definition** **8.** *(Candidate Destination): Given a service s_i_ on a PDSG, a reachable service s_j_ becomes a candidate destination of the service events generated by s_i_ if there is no reachable service s_j_’ (s_j_’ ≠ s_j_), which s_j_ is on a path from s_i_ to s_j_’.*

The candidate destination set of a service is the all reachable services from this service, whose out-degree is 0. The main task of getting *s_i_*’s candidate destination set is to generate all reachable services of *s_i_*. This can be achieved as follows: Each graph has a reachable matrix to reflect its reachability. A PDSG corresponds to a reachable matrix M_n__*n_, where n is the service number on the PDSG, and the element *M*[*i*, *j*] at the *i*th line *j*th column is 1, if *s_i_* reaches *s_j_*; otherwise, *M*[*i*, *j*] = 0. The candidate destination set of an arbitrary service *s_i_* can be expressed as *CDS*(*s_i_*) = {*s_j_*|*M*[*i*,*j*] = 1 ∧ *d_out_*(*s_j_*) = 0}, where *d_out_*(*s_j_*) is the out-degree of *s_j_*.

A service *s_i_* may reach a candidate destination via multiple paths. It has to route a service event on the most probable path to this candidate destination. It means, a service should select the target service pointing by its hyperlinks which will most probably route the service event into the candidate destination. We develop a heuristic approach based on A* algorithm to help a service make a selection automatically. Our approach considers the heuristic that estimates the most probable path to each candidate destination, which means to maximize *f* = *g* + *h*, *g* is the probability from service *s_i_* to an arbitrary service, *h* is the probability from the arbitrary service to a candidate destination. In this paper, the occurrence of a service event is only related to its casual service events’ occurrence. Under this case, we can calculate *h* by multiply the probabilities on a path from the arbitrary service to the candidate destination.

The above algorithm can route a generated service event to the most probable destination along the most probable path. To avoid an endless routing of a service event, we have to discuss when a routing path should be terminated. From the algorithm, we get that a path is terminated if any service except for the candidate destination on this path has no target services. Besides, a routing path will also be terminated when the effect event does not occur during the time interval labelled on the corresponding edge. There are two cases: If the effect event does not occur at all, the path is stopped; If it occurs beyond the time interval, the effect event is considered as a new service event need to be routed. This event and its service are input into our algorithm to select target services for routing.

**Figure 4:**
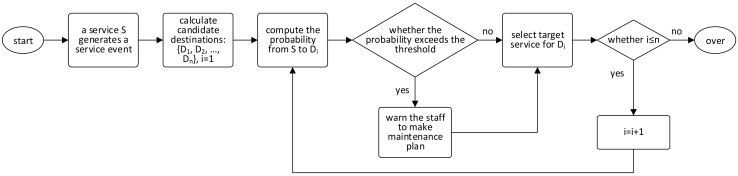
Workflow of our predictive industrial maintenance approach.

Based on the event routing approach presented above, we put forward a novel predictive industrial maintenance approach. Figure 4 illustrates the workflow of our approach. When an edge/cloud service *s_i_* generates a service event, it will compute its candidate destination set *CDS*(*s_i_*) = {*d*_1_, *d*_2_, …, *d_n_*}. For each candidate destination *d_j_*, service *s_i_* computes the probability from itself to *d_j_*. If the probability is no less than a predefined probability threshold, *s_i_* will make a warning to the staff for making maintenance plan of the related fault. Whether the probability exceeds the threshold or not, service *s_i_* will select the target service for the most probable path from *s_i_* to *d_j_* for routing the generated service event. After this, the process is over. Any service generating a service event will start a new process same with this one.

## 7. Proactive Data Service Graph Validation

This section validates the completeness of the approach by model checking techniques. We firstly introduce the symbolic transition system to model an industrial system.

A symbolic transition system can be a formal description of industrial systems. It is used for modeling the system state behaviors, i.e., how a system goes from state to state. A symbolic transition system *S* can be defined as a three tuple *S* = 〈*X*, *I*, *T*〉, where *X* is a non-empty set of system state variables, *I* is the initial states of *S*, and *T* is the transition relation between states and next states. Domain of a variable *x* ∈ *X* is denoted as *D*(*x*). A state *s* of *S* is an assignment to the state variables *X*. All possible states of *S* are denoted as *P*(*S*). There is a variable *τ*∈*X* with *D*(*τ*) = R^+^ (the set of non-negative real numbers) representing the timestamp of each state, i.e., an assignment to *X* - {*τ*}. Thus, a trace *π* of *S* is an infinite time-ordered sequence of states denoted as *π* = *s*_0_, *s*_1_, …, *s_i_*, …, where *s*_0_ ⊨ *I*, and ∀*k* ≥ 0, (*s_k_*, *s_k_*_+1_) ⊨ *T*. ⊨ is the satisfaction relation representing a variable assignment satisfies a formula, i.e., the formula is true under the variable assignment. The *k*th state of a trace *π* is written *π*[*k*].

Based on the above concepts, an industrial system can be described as a symbolic transition system. In order to interpret how the states of this system change over time, metric temporal logic is introduced below.

Metric temporal logic (MTL) is a timed extension of linear temporal logic (LTL). Given a set APs of atomic propositions, the formulae of MTL are built on APs by Boolean connectives and temporal operators as *φ*::= ⊥|⊤|*p*|¬*φ*|*φ∧ϕ*|*φ∨ϕ*|*φU_I_ϕ*|*φS_I_ϕ*|♢*_I_φ*|◻*_I_φ*, where ⊥ represents false, ⊤ represents true, and *p*∈*APs*. *U_I_*, *S_I_*, ♢*_I_* and ◻*_I_* are temporal operators, in which *I* is an interval as [*a*, *b*], [*a*, *b*), (*a*, *b*], (*a*, *b*), *a*, *b*∈R^+^∪{+∞}. *U_I_* is a time-constrained *until* operator, and *φU_I_ϕ* means *φ* will be true lasting a time interval no longer than *I* until a time when *ϕ* is true. *S_I_* is a time-constrained *since* operator, and *φS_I_ϕ* means *φ* has been true lasting a time interval no longer than *I* since a time when *ϕ* was true. ♢*_I_* is a time-constrained *eventually* operator, and ♢*_I_φ* means *φ* will be true at some future time, where the time interval during which *φ* is not true, is no longer than *I*. ◻*_I_* is a time-constrained *always* operator, and ◻*_I_φ* means *φ* will be true lasting a time interval no longer than *I* in the future.

**Figure 5:**
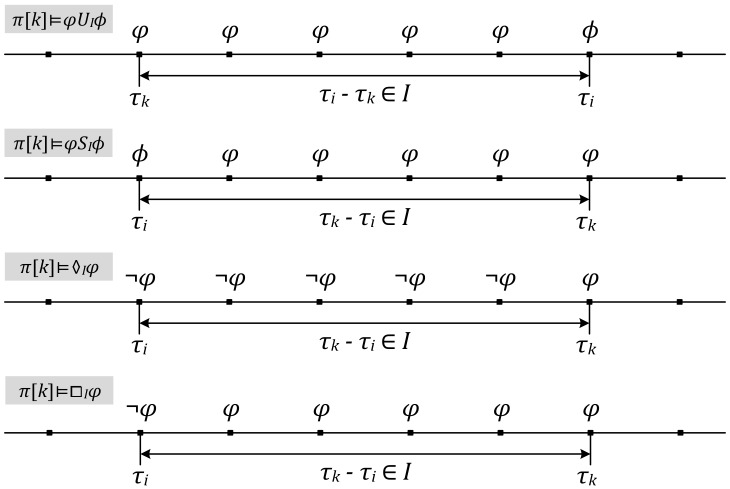
Illustration of *π*[*k*] Satisfying Some Metric Temporal Logic (MTL) Formulae.

Given a symbolic transition system *S*, *π* is a trace of *S*. The *k*th state of *π*, *π*[*k*], satisfies an MTL formula *φ* can be categorized as follows. Figure 5 illustrates the last four expressions.

- *π*[*k*] ⊨ *p*, if and only if *p* is an atomic proposition which is true under *π*[*k*].
- *π*[*k*] ⊨ ¬*φ*, if and only if not *π*[*k*] ⊨ *p*.
- *π*[*k*] ⊨ *φ*∧*ϕ*, if and only if *π*[*k*] ⊨ *φ* and *π*[*k*] ⊨ *ϕ*.
- *π*[*k*] ⊨ *φ*∨*ϕ*, if and only if *π*[*k*] ⊨ *φ* or *π*[*k*] ⊨ *ϕ*.
- *π*[*k*] ⊨ *φU_I_ϕ*, if and only if ∃*i* > *k*, *π*[*i*] ⊨ *ϕ*, *τ_i_*-*τ_k_* ∈ *I*, ∀*k* ≤ *j* < *i*, *π*[*j*] ⊨ *φ*.
- *π*[*k*] ⊨ *φS_I_ϕ*, if and only if ∃*i* < *k*, *π*[*i*] ⊨ *ϕ*, *τ_k_*-*τ_i_* ∈ *I*, ∀*i* < *j* ≤ *k*, *π*[*j*] ⊨ *φ*.
- *π*[*k*] ⊨ ♢*_I_φ*, if and only if ∃*i* < *k*, *π*[*k*] ⊨ *φ*, *τ_k_*-*τ_i_* ∈ *I*, ∀*i* ≤ *j* < *k*, *π*[*j*] ⊨ ¬*φ*.
- *π*[*k*] ⊨ ◻*_I_φ*, if and only if ∃*i* < *k*, *π*[*i*] ⊨ ¬*φ*, *τ_k_*-*τ_i_* ∈ *I*, ∀*i* < *j* ≤ *k*, *π*[*j*] ⊨ *φ*.

To map an industrial system trace into an event routing path on a PDSG, we define a PDSG as a description of industrial systems.

**Definition** **9.** *(Proactive Data Service System, PDSS): Given a PDSG, a PDSS is a three-tuple S_G_ = 〈X_G_, I_G_, T_G_〉, where X_G_ = ES∪CS∪{τ}, ∀x_g_∈X_G_, D(x_g_) = {⊥, ⊤}, and ES, CS are the edge and cloud services on the PDSG respectively; I_G_ = X∧(τ = 0); T_G_ = ∧_(x__g__∈ES__∪CS)_(x_g_→x_g_’)∧(τ≤τ’)∧((∨_(x__g__∈ES__∪CS)_(x_g_ ≠ x_g_’))→(τ = τ’)), x_g_’ is the next state of x_g_.*

The definition of *T_G_* reflects the assumption that a state can last for a period but change instantly. To describe system state behaviors, a state variable *x_g_*∈*ES* of a PDSS will be expressed as a set of predicates. These predicates are generated according to the preset operations in each edge service. For example, BT sensor data can be expressed as {*d_BT_*.*value* ≤ 20, 20 < *d*_BT_.*value* < 80, *d_BT_*.*value* ≥ 80}. It is because the preset operations (Some classification-based outlier detection method will classify the data and consider the classes with small data as outliers.) classify the input sensor data into three classes, in which data *d* satisfying *d*.*value* ≤ 20 and *d*.*value* ≥ 80 are outliers. Formally, each state variable *x* ∈ *X* in an industrial system model *S* is mapped into a state variable *x_g_* in a PDSS. It is expressed by a non-empty set *p_x_* of predicates. The mapping function is denoted as *M*. In this way, an assignment to a state variable can be expressed as a proposition. Therefore, system states and traces of *S* can be expressed as MTL formulae. Herein, a trace *π* of *S* is mapped into a routing path of a PDSS, which is denoted as *π*’.

Next, we discuss how to judge *π*’ satisfies the constraints of the corresponding PDSG. The main constraints are the two types of each cloud service and the time interval on edges pointing to the service.

A routing path *π*’ of a PDSS satisfies an OR node, if and only if any state *π*’[*k*] of *π*’ holds the following conditions: (1) If *π*’[*k*] satisfies an OR node *f_or_*, then a consecutive set of states *π*’[*j*], *π*’[*j* + 1], …, and *π*’[*k*] satisfied *f_or_*, and there is a time interval left adjacent to *π*’[*j*], states in which satisfied at least one node *v*, where (*v*, *f_or_*) is an edge pointing to *f_or_*; (2) Any node n satisfying condition 1) also satisfies that its corresponding interval does not exceed the propagation time interval *T_int_* labelled on the edge (*v*, *f_or_*).

Similarly, a routing path *π*’ of a PDSS satisfies an AND node, if and only if any state *π*’[*k*] of *π* holds the following conditions: (1) If *π*’[*k*] satisfies an AND node *f_and_*, then a consecutive set of states *π*’[*j*], *π*’[*j* + 1], …, and *π*’[*k*] satisfied *f_and_*, and for each node *v* pointing to *f_and_* there is a time interval *I*^(*v*)^ left adjacent to *π*’[*j*], states in which satisfied *v*; (2) For any node *v* satisfying condition (1), its corresponding interval *I*^(*v*)^ does not exceed the propagation time interval *T_int_* labelled on the edge (*v*, *f_and_*).

**Figure 6:**
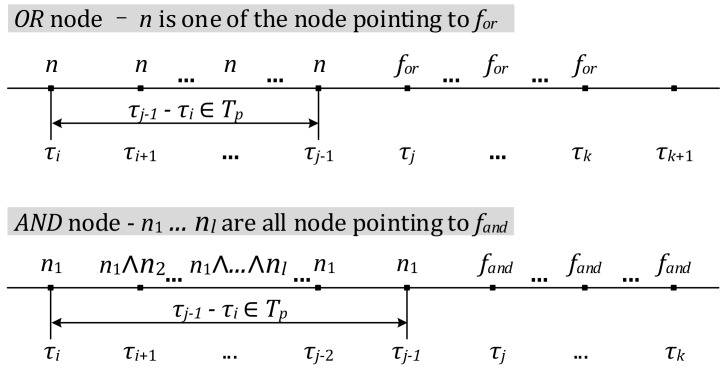
Conditions for a trace *π*’ of a PDSS to satisfying an OR/AND node on a PDSG.

Figure 6 illustrates the conditions for satisfying an OR node and an AND node. These time-constrained conditions can be described by MTL formulae with temporal operators.

If a routing path *π*’ of a PDSS satisfies all OR and AND nodes on the corresponding PDSG, *π*’ satisfies the PDSG. All system traces of S are mapped into paths *Π*’ in the PDSS. If each event routing path in *Π*’ satisfies the PDSG, we suggest that any system state behavior which exists in the industrial system can be captured by the graph.

## 8. Results

### 8.1. Experiment Setup

Datasets: The following experiments use a real sensor dataset from a coal power plant. The dataset contains sensor data from 2014-10-01 00:00:00 to 2015-04-30 23:59:59. Totally 182 sensors deployed on 5 interactive devices are involved. Each sensor generates one piece of data per second. We divide the set into two parts. The training set is from 2014-10-01 00:00:00 to 2015-03-31 23:59:59. The testing set is from 2015-04-01 00:00:00 to 2015-04-30 23:59:59.

The first part of our experiments is to test the effectiveness of our approach. We observe the variation of the service event correlation number and service hyper-link number under the rise of dataset scale. We also analyze that how the dataset scale affects the effectiveness of our approach. The maintenance records of this plant from 2014-10-01 00:00:00 to 2015-04-30 23:59:59 are used to verify the effectiveness. There are 48 and 9 maintenance records during the time range of the training set and the testing set respectively. Notably, we only consider the records with faults occurring both in training set and testing set. On the other hand, we compare the effects on early warnings of maintenance between our approaches and three typical approaches. The second part of our experiments is to test the performance of our approach under edge computing paradigm and cloud computing paradigm.

Baselines: We select one predictive industrial maintenance solution used in practice and two typical data-driven anomaly detection approaches as our baselines. The solution is the range-based approach, which customizes value bounders for individual sensors based on inspectors’ experiences, sensor/device instructions. The other two approaches are COF outlier detection approach [50] and Matrix Profile discord discovery approach [51]. As Related Works Section summarizes, such approaches can be applied in predictive industrial maintenance from the perspective of individual sensors. We do not choose any model-based approaches as we have no enough prior knowledge about a real power plant.

Environments: The experiments are done on a PC with four Intel Core i5-2400 CPUs 3.10 G Hz and 4.00 GB RAM. The operating system is Windows 7 Ultimate. All the algorithms are implemented in Java with JDK 1.8.0.

### 8.2. Effectiveness

#### 8.2.1. Effects of Our Approach

##### Variation of Correlation Number and Hyperlink Number

**Figure 7:**
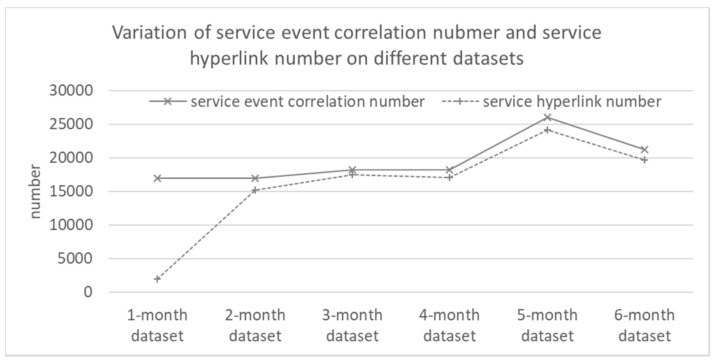
Variation of correlation number and hyperlink number on different datasets with *p* ≥ 0.8.

Our training set spans six months, including October, November, December 2014 and January, February and March 2015. This part of experiments tries to verify how the correlation number and hyperlink number changes on a 1-month (October 2014), 2-month (October and November 2014), …, 6-month (the whole training set) dataset. This experiment encapsulates the service event correlations with no less than 0.8 probability (i.e., *δ_p_* = 0.8) into service hyperlinks. We record the results and draw them in Figure 7.

As shown in Figure 7, in most cases, both of the correlation number and the hyperlink number increase with the rise of input dataset scale. On the other hand, there is an exception for the 6-month dataset. The number drops. It partially reveals that the number will not increase without limit. We will conduct in-depth research in our future work. Besides, the service hyperlink number is close to the service event correlation number except on the 1-month dataset. It is because a smaller dataset contains many occasional service event correlations.

##### Effectiveness of Our Approach

In this experiment, sensor data in the testing set are input into corresponding service in form of stream. By a sliding window, each service detects service events from each sensor data sequence in the current sliding window. A service judges whether it should make a warning of maintenance and selects which target service it should route the generated service event into. After all streams simulated from the testing set are processed, we count the warning results to analyze the effectiveness. Details of the process can be found in Section 6.

To measure the effectiveness, we use the following indicators. Precision is the number of correct results divided by the number of all results. Recall is the number of correct results divided by the number of results that should have been returned.

**Figure 8:**
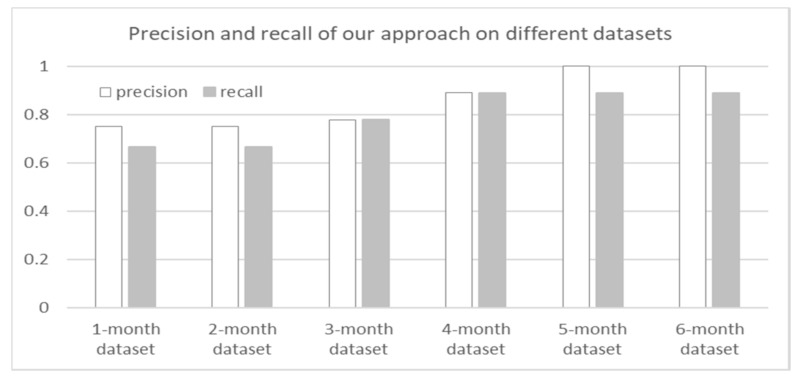
The precision and recall of our approach on different datasets.

Final results are drawn in Figure 8. As shown in Figure 8, our precision and recall both show a growing trend with the rise of dataset scale. It is because our approach makes more correct warnings on a larger dataset. But the results’ number of our approach increases firstly and then drops. At first, a small dataset (1-month and 2-month dataset) contains a few faults so that our approach makes a few warnings of maintenance. Thus, on 3-month and 4-month dataset, our approach makes more warnings since the datasets contain more faults. However, when the dataset becomes larger, more service hyperlinks are generated. It can help reduce false positives. Consequently, on the 5-month and 6-month dataset, our approach outputs less but correct results. It causes that the precision is higher than the recall on the last two datasets.

Actually, we also compare the effects of our approach with edge computing paradigm and cloud computing paradigm. The results show that the paradigm doesn’t have obvious impacts on the effects of predictive industrial maintenance.

This part of experiments (Section 8.2.1) shows that, larger scale of dataset will generate much more service hyperlinks. And more service hyperlinks lead to more precision and recall. However, obviously, huge service hyperlinks will improve the complexity of our event routing algorithm. In our future work, we plan to research that whether we can sacrifice tolerable effectiveness to abandon some service hyperlinks.

#### 8.2.2. Comparative Effects of Different Approaches

In this part, we make warnings of maintenance by our approach and other three typical approaches.

To make predictive industrial maintenance, we mine the association rules [45] between anomalies and recorded faults in maintenance records. The anomalies associated with the recorded faults are listed in Table 2.

Once the associated anomalies are detected by the range-based approach, outlier detection approach or discord discovery approach, they will make a warning of maintenance for the corresponding fault. Based on this, we compare the warning time between our approach and the other approaches. Warning time is the difference between the timestamp an approach makes a warning of maintenance for a fault and the starting time of this fault.

Table 3 presents the final results. As this table shows, our approach makes warnings earliest in the four approaches for each fault. Generally, the shortest warning time appears in the range-based approach. The reason is that a range is usually a threshold for a significant fault in a device. If sensor data exceed a range, the fault does probably happen immediately. Besides, some faults are formed by several anomalies without exceeding the range. Thus, it failed to make early warnings for most times.

It is possible not to be able to make a warning of maintenance by the outlier detection approach and dis-cord discovery approach either. We look into the middle results to analyze the reasons. Sometimes sensor data ascend/descend gradually. The outlier detection approach cannot detect such abnormal behaviors. On the other hand, if a sensor data sequence has similar subsequences, such as sudden drop, the discord discovery approach will not identify such subsequences as the most dissimilar ones.

Table 3 presents that sometimes the outlier detection approach and discord discovery approach have same warning time. The reason is that the two approaches can detect same faults sometimes. For example, the two approaches can both identify the over low coal feed fault in Figure 1.

Besides, the discord discovery approach tends to have a longer warning time than the outlier detection approach. It is because that this approach may discover a subsequence, which contains an outlier. In this case, it will make a warning ahead of the outlier detection approach.

Based on the comparative analysis, our correlation driven event routing approach cannot only predict warnings more effectively than the typical approaches, but also predict them more earlier. Thus, our approach can make maintenance plans more effectively and earlier to avoid loss. It verifies that our approach has some practical significance. However, owing to the lack of prior knowledge, we do not test any model-based approaches on our power plant dataset. In this case, we cannot compare our approach with model-based approaches. In our future work, we will learn more prior knowledge from inspectors’ experiences and investigate more predictive maintenance approaches designed for power plants and reproduce them for comparing with ours. We will further improve our approach based on the comparative analysis.

### 8.3. Efficiency

We further test our approach’s performance under edge computing paradigm and cloud computing paradigm [5,6] with different data source numbers, i.e., number of physical sensors. We use the following indicator.

**Definition** **10.** *(Average Latency): Let t_i_ is the time our algorithm consumes to route the ith output service event. Let N be the current size of all output service events, the average latency of an approach can be defined as t_lat_ = ∑_i_t_i_/N.*

**Figure 9:**
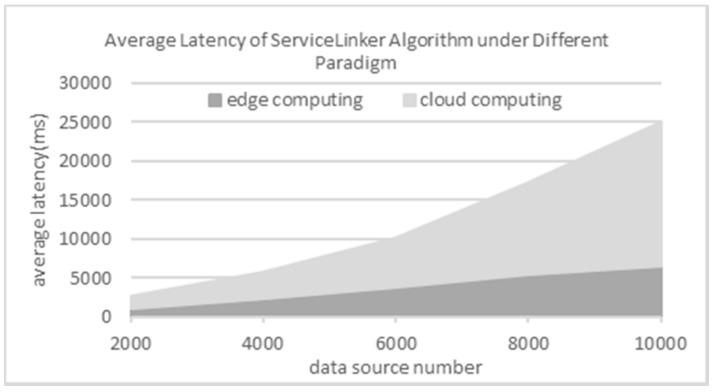
Average latency under edge computing and cloud computing on different synthetic datasets.

In this part, we experiment with the five synthetic datasets containing 2000, 4000, …, 10,000 data sources respectively. Herein, each dataset with k data sources is simulated to be k streams with one records per second. The results are shown in Figure 9. As this figure shows, the average latency increases linearly with the growth of data source number under edge computing paradigm. On the other hand, the average latency increases exponentially under cloud computing paradigm. It verifies that the approach in this paper can effectively reduce the average latency when routing service events for predictive industrial maintenance.

## 9. Conclusions

Predictive industrial maintenance is a hot topic in mechanical engineering. Existing predictive industrial maintenance techniques usually construct a model based on prior knowledge or data in the build-time. This paper makes an attempt on predicting anomalies/faults propagation by using the correlations among anomalies/faults. We map the anomalies/faults propagation into event routing among services via service hyperlink based on our previous work and propose a correlation driven event routing algorithm to perform predictive industrial maintenance. To reach our goal, we have to generate service hyperlinks firstly. This paper proposes an effective algorithm to discover causal relationships among anomalies/faults and encapsulate them into service hyperlinks. Based on the generated service hyperlinks, a heuristic event routing approach is proposed to handle the uncertainty problem. We also verify the completeness of our approach by model checking techniques to guarantee the effectiveness of our approach in theory. Besides, we refine our proactive data service model to enable our approach to be applied in information-sensitive industrial enterprises in practice. Experiment results show that our approach can reach 100% precision and 88.89% recall at most. However, the large scale of service hyperlinks can improve the effectiveness of our event routing algorithm and reduce the efficiency. Our future work will try to balance the effectiveness and efficiency, which means sacrifice tolerable effectiveness to improve efficiency. On the other hand, learn more prior knowledge to reproduce some model-based predictive industrial maintenance approaches. In this way, we can future improve our approach based on the comparative analysis.

## Figures and Tables

**Table 1:** Abbreviations.

	Abbreviation	Explanation
**device**	CFD	coal feeder
	CM	coal mill
	PAF	primary air fan
**sensor/service**	AP	active power
	BT	bear temperature
	CAVD	cold air valve degree
	CF	coal feed
	DPGB	differential pressure of grinding bowl
	DPSF	differential pressure of strainer filter
	E	electricity
	HAVD	hot air valve degree
	IAP	inlet air pressure
	IPAP	inlet primary air pressure
	IPAT	inlet primary air temperature
	IPAV	inlet primary air volume
	OTT	oil tank temperature
	UL	unit load
	V	vibration
**anomaly/fault/event type**	CB	coal blockage
	CI	coal interruption
	H-CAVD	over high cold air valve degree
	H-DPSF	over high differential pressure of strainer filter
	H-HAVD	over high hot air valve degree
	H-IPAT	over high inlet primary air temperature
	H-V	over high vibration
	L-AP	over low active power
	L-BT	over low bear temperature
	L-CF	over low coal feed
	L-DPGB	over low differential pressure of grinding bowl
	L-E	over low electricity
	L-HAVD	over low hot air valve degree
	L-IAP	over low inlet air pressure
	L-IPAP	over low inlet primary air pressure
	L-IPAT	over low inlet primary air temperature
	L-IPAV	over low inlet primary air volume
	L-OTT	over low oil tank temperature
	L-UL	over low unit load

**Table 2:** Faults and its associated events.

Fault Type		Associated Anomalies	Conf ^1^
L-IPAV fault on a PAF device	*AE*_1_^2^	L-IPAT, L-HAVD, L-IPAP.	100.00%
	*AE*_2_	L-E on CM.	100.00%
	*AE*_3_	L-IPAT, L-IPAP.	80.00%
L-IPAP fault on a PAF device	*AE*_1_	H-CAVD, L-OTT.	86.96%
CB fault on a CM device	*AE*_1_	H-HAVD, L-IAP.	100.00%
	*AE*_2_	L-IPAT.	88.89%
H-DPSF fault on a CM device	*AE*_1_	L-BT on PAF.	100.00%

^1^ ‘Conf’ is the confidence of an association rule; ^2^ ‘*AE_i_*’ is the *i*th set of associated events of a fault.

**Table 3:** Warning time of different approaches (unit: min).

	Fault Type	L-IPAV			L-IPAP	CB		L-DPSF
Approaches		*AE*_1_	*AE*_2_	*AE*_3_	*AE*_1_	*AE*_1_	*AE*_2_	*AE*_1_
**Our Approach**		70	58	82	152	63	96	132
**Range-based Approach**		- ^1^	12	9	-	15	2	-
**Outlier Detection Approach**		18	21	-	31	23	19	33
**Discord Discovery Approach**		-	21	19	31	35	26	34

^1^ ‘-’ represents this approach cannot make a warning.
